# Trajectories of antidepressant use before and after a suicide attempt among refugees and Swedish-born individuals: a cohort study

**DOI:** 10.1186/s12939-021-01460-z

**Published:** 2021-06-02

**Authors:** Ridwanul Amin, Syed Rahman, Magnus Helgesson, Emma Björkenstam, Bo Runeson, Petter Tinghög, Lars Mehlum, Ping Qin, Ellenor Mittendorfer-Rutz

**Affiliations:** 1 Division of Insurance Medicine, Department of Clinical Neuroscience, Karolinska Institutet, SE-171 77 Stockholm, Sweden; 2 Centre for Psychiatry Research, Department of Clinical Neuroscience, Karolinska Institutet, Stockholm County Council, SE-112 81 Stockholm, Sweden; 3 Swedish Red Cross University College, Hälsovägen 11, SE-141 57 Huddinge, Sweden; 4 National Centre for Suicide Research and Prevention, University of Oslo, 0374 Oslo, Norway

**Keywords:** Migration, Refugees, Suicide attempt, Antidepressant, Trajectory

## Abstract

**Background:**

To identify key information regarding potential treatment differences in refugees and the host population, we aimed to investigate patterns (trajectories) of antidepressant use during 3 years before and after a suicide attempt in refugees, compared with Swedish-born. Association of the identified trajectory groups with individual characteristics were also investigated.

**Methods:**

All 20–64-years-old refugees and Swedish-born individuals having specialised healthcare for suicide attempt during 2009–2015 (*n* = 62,442, 5.6% refugees) were followed 3 years before and after the index attempt. Trajectories of annual defined daily doses (DDDs) of antidepressants were analysed using group-based trajectory models. Associations between the identified trajectory groups and different covariates were estimated by chi^2^-tests and multinomial logistic regression.

**Results:**

Among the four identified trajectory groups, antidepressant use was constantly low (≤15 DDDs) for 64.9% of refugees. A ‘low increasing’ group comprised 5.9% of refugees (60–260 annual DDDs before and 510–685 DDDs after index attempt). Two other trajectory groups had constant use at medium (110–190 DDDs) and high (630–765 DDDs) levels (22.5 and 6.6% of refugees, respectively). Method of suicide attempt and any use of psychotropic drugs during the year before index attempt discriminated between refugees’ trajectory groups. The patterns and composition of the trajectory groups and their association, discriminated with different covariates, were fairly similar among refugees and Swedish-born, with the exception of previous hypnotic and sedative drug use being more important in refugees.

**Conclusions:**

Despite previous reports on refugees being undertreated regarding psychiatric healthcare, no major differences in antidepressant treatment between refugees and Swedish-born suicide attempters were found.

**Supplementary Information:**

The online version contains supplementary material available at 10.1186/s12939-021-01460-z.

## Introduction

Despite the strong association between mental ill-health and suicidal behaviour, a vast majority of people with mental disorders do not die by suicide [1]. Therefore, identification of additional risk factors within vulnerable groups such as refugees is important because refugees were reported to have higher levels of mental disorders such as depressive or anxiety disorder and post-traumatic stress disorder (PTSD) [2–5] but lower rates of suicide attempt [6–8], in comparison with the general population in their respective host country. In this regard, healthcare contacts and related medical treatment before a suicide attempt may offer opportunities for prevention of suicidal behaviour. Similarly, contact with healthcare professionals and adequate treatment follow-up after a suicide attempt provide the possibility of treatment intervention to obtain favourable outcomes concerning repeated attempts and suicide. Concerning healthcare use and treatment in general and in suicide attempters, refugees were found to be disadvantaged, in comparison with their host population [9].

Language barriers, stigma associated with reporting of mental ill-health, inadequate knowledge on how to access healthcare and possible lack of cultural competence among healthcare professionals were suggested as factors contributing to lower treatment rates in refugees [5, 9]. Antidepressants are the most commonly prescribed psychiatric medications [10] and therefore, the patterns/trajectories of antidepressant use, before and after a suicide attempt among refugees may reveal key information regarding treatment for affective and anxiety disorders commonly seen in suicide attempters. To the authors’ best knowledge, such patterns of antidepressant use have never been investigated in refugee suicide attempters.

The use of psychotropic drugs (including antidepressants) in refugees might be influenced by sex [11], labour market marginalisation [12] and clinical characteristics such as the underlying mental disorders [13]. It is likely that antidepressant use before and after a suicide attempt in refugees is influenced by these factors. Therefore, it is important to investigate to which extent such characteristics are related to the identified patterns in refugees and identify potential differences between refugees and Swedish-born attempters.

### Aims

We aimed 1) to investigate the patterns (trajectories) of antidepressant use in refugees and the Swedish-born population, during an observation window of 3 years before and 3 years after a suicide attempt and, 2) to elucidate if the identified trajectory groups of antidepressant use are associated with socio-demographic, labour market marginalisation, migration-related and clinical factors among refugees in Sweden, compared with the Swedish-born.

## Materials and methods

### Study population

An open cohort of all individuals treated for suicide attempt in inpatient or specialised outpatient healthcare in Sweden during January 1st 2009 - December 31st 2015 and 20–64 years of age at the date of attempt was identified (*n* = 72,306 suicide attempters). In case of repeated attempts during the follow-up, the chronologically first attempt was considered as the index attempt. As we intended to measure annual use of antidepressants during the 3-years before the index attempt, non-residents were excluded (*n* = 1806). Furthermore, individuals with missing data regarding their country of birth and reason for residence in Sweden (*n* = 18 and *n* = 2477, respectively) were excluded. Non-refugee migrants (*n* = 5563) were not included. The final study population comprised 3492 (5.6%) refugees and 58,950 (94.4%) Swedish-born individuals.

### Refugees and the Swedish-born population

The Swedish Migration Agency grants residence permits to refugees according to the Geneva Convention definition [14]. Additionally, individuals granted residence permits due to ‘in need of protection’ or ‘humanitarian grounds’ as their ‘reason for residence’ are also considered as refugees by the Swedish Migration Agency [15]. In this study, refugee status was measured in the year before the suicide attempt according to the criteria used by the Swedish Migration Agency. People born in Sweden were identified as ‘Swedish-born’ in this study.

### Registers

Suicide attempts were identified from the National Patient Register [16, 17]. By using de-identified personal identification numbers, data linkage was done at an individual level from the following registers:

1) Statistics Sweden: LISA database (Longitudinal integration database for health insurance and labour market studies) [18] containing data on sex, age, country of birth, educational level, family situation, type of residential area, number of annual net days with sickness absence benefits, disability pension and number of annual days with unemployment; STATIV database (Longitudinal database for integration studies) containing data on reason for residence (e.g. refugee).

2) National Board of Health and Welfare: i) National Patient Register containing data on date and diagnosis of inpatient and specialised outpatient healthcare; ii) Prescribed Drug Register [19] containing information on prescribed and dispensed drugs, dates, dosages, amount and Anatomical Therapeutic Chemical (ATC) Classification System code; iii) Cause of Death Register [20] containing data on date and cause of death.

### Suicide attempts

The International Classification of Diseases version 10 (ICD-10) codes for intentional self-harm and events of undetermined intent (ICD-10 code X60-X84 and Y10-Y34, respectively) were used to identify suicide attempts. The inclusion of events of undetermined intent is analogous with a method used in previous studies, which reduced bias from underreporting and variations in case ascertainment in different regions and time periods [21–23]. A sensitivity analysis, excluding individuals with undetermined intent, was done to test the comparability of the results (*n* = 2027 refugees, 32,654 Swedish-born were included). Another sensitivity analysis was conducted excluding individuals with specialised outpatient healthcare due to suicide attempt to check if the results are comparable with the main analysis (*n* = 1635 refugees, 27,122 Swedish-born were included).

### Outcome

The ATC code N06A was used to identify the antidepressants. Annual use of antidepressants, measured as the level of defined daily dose (DDD) [24], during the 3 years before and after the index attempt was assessed. An annual time scale was used, with t0 indicating the date of index attempt, Y-1 to Y-3 referring to the three respective years before t0, and Y + 1 to Y + 3 referring to the three respective years after t0. The sum of the DDDs of all antidepressants prescribed to an individual during a given year indicates the total amount of DDDs for that individual for that specific observation year (in a sensitivity analysis, antidepressant use was also measured 6-monthly during the follow-up period among refugees, the Swedish-born individuals and the whole cohort). Any annual DDD > 1500 was considered unusual (e.g. error in data or large purchase before travel) and truncation was done for DDDs that exceeded that level. Outcome data was available until 31 October 2018 and therefore, data on antidepressant use was lacking for 0.02% of refugees and Swedish-born individuals for the last 2 months during Y + 3.

### Covariates

To explore how much of the variability of the identified trajectory groups of antidepressant use is explained by different individual-level characteristics, the following factors were considered as potential covariates: A. socio-demographic factors (sex, age, educational level, family situation and type of residential area); B. Labour market marginalisation factors (unemployment, sickness absence, disability pension); C. Clinical factors (history of any specialised healthcare due to suicide attempt and history of any specialised healthcare due to somatic diagnoses during 3 years before index attempt, method of index attempt, specific mental disorder as main or secondary diagnosis in specialised healthcare at index attempt, history of psychotropic drug use (annual DDDs of neuroleptics, anxiolytics and sedatives with ATC codes N05A, N05B and N05C, respectively); D. Migration-related factors (country of birth, duration of residence; for refugees only). All factors were measured during the year before index attempt if not otherwise stated. Missing values for a covariate were categorised separately. Table 1 shows the categorisation of the socio-demographic, labour market marginalisation and clinical factors.

**Table 1:** Socio-demographic, labour market marginalisation and clinical factors among the study population^a^

Characteristics	Swedish-born n (column %)	Refugees n (column %)
*Socio-demographic factors*^*b*^		
Sex		
Women	27,958 (47.4)	1573 (45.0)
Men	30,992 (52.6)	1919 (55.0)
Age (years)		
20–24	13,351 (22.6)	580 (16.6)
25–34	14,134 (24.0)	1028 (29.4)
35–44	11,278 (19.1)	865 (24.8)
45–54	11,548 (19.6)	714 (20.4)
55–64	8639 (14.7)	305 (8.7)
Educational level (years)		
Compulsory school (0–9)	15,226 (25.8)	1221 (35.0)
High school (10–12)	31,830 (54.0)	1358 (38.9)
College or university (> 12)	11,295 (19.2)	808 (23.1)
Missing	599 (1.0)	105 (3.0)
Family situation		
Married/cohabiting without children living at home	6200 (10.5)	366 (10.5)
Married/cohabiting with children living at home	9544 (16.2)	948 (27.1)
Single/divorced/separated/widowed without children living at home	39,533 (67.1)	1860 (53.3)
Single/divorced/separated/widowed with children living at home	3673 (6.2)	318 (9.1)
Type of residential area^c^		
Big cities	18,680 (31.7)	1705 (48.8)
Medium-sized cities	21,395 (36.3)	1173 (33.6)
Small cities/villages	18,875 (32.0)	614 (17.6)
*Labour market marginalisation factors*^b, d^		
Unemployed, 1–180 days	9486 (16.1)	770 (22.1)
Unemployed, > 180 days	2015 (3.4)	326 (9.3)
Sickness absence, 1–90 net days	5955 (10.1)	242 (6.9)
Sickness absence, > 90 net days	5267 (8.9)	240 (6.9)
Disability pension	11,332 (19.2)	437 (12.5)
*Clinical factors*		
History of any suicide attempt^e^	3868 (6.6)	100 (2.9)
Method of index attempt (ICD-10 code^f^)		
Self-poisoning (X60–69, Y10–19)	28,365 (48.1)	1793 (51.3)
Self-injury (X70–84, Y20–34)	9336 (51.9)	1699 (48.7)
Mental disorder^g^ at index attempt (ICD-10 code^f^)		
Depressive disorders (F32-F34)	3164 (5.4)	249 (7.1)
Bipolar disorders (F30-F31)	815 (1.4)	15 (0.4)
Anxiety disorders (F38-F48 except F43.1)	2771 (4.7)	169 (4.8)
Post-traumatic stress disorder (F43.1)	81 (0.1)	40 (1.1)
Schizophrenia, schizotypal and delusional disorder (F20-F29)	557 (0.9)	46 (1.3)
Other mental disorders (F01-F19, F50-F99)	8260 (14.0)	237 (6.8)
History of specialised healthcare use due to somatic diagnoses^h^	44,948 (76.2)	2658 (76.1)
Use of psychotropic drug(s)^i^ except antidepressants		
Neuroleptic drug(s) (N05A)^j^	8634 (14.6)	466 (13.3)
Anxiolytic drug(s) (N05B)^j^	18,105 (30.7)	751 (21.5)
Hypnotic and sedative drug(s) (N05C)^j^	20,183 (34.2)	997 (28.6)
Use of antidepressants^i^ (N06A)^j^	25,525 (43.3)	1393 (39.9)
Index attempts		
From inpatient healthcare	27,122 (46.0)	1635 (46.8)
From specialised outpatient healthcare	31,828 (54.0)	1857 (53.2)

Differences between the Swedish-born individuals and refugees regarding all socio-demographic, labour market marginalisation and clinical factors were statistically significant based on χ2 tests (*p* < 0.05)

^a^Study population: 58,950 Swedish-born and 3492 refugees, aged 20–64 years and residing in Sweden who sought inpatient or specialised outpatient healthcare due to a suicide attempt (index attempt) in between 2009 and 2015 in Sweden

^b^All socio-demographic and labour market marginalisation factors were measured during the year before index attempt except sex and age which were measured at the index attempt

^c^Type of residential area: big cities - Stockholm, Gothenburg and, Malmö; medium-sized cities - cities with more than 90,000 inhabitants within 30 km distance from the centre of the city; small cities/villages

^d^‘No unemployment’, ‘No sickness absence’ and ‘No disability pension’ categories are not presented

^e^Measured as any inpatient or specialised outpatient healthcare due to suicide attempt during the 3 years before index attempt

^f^International Classification of Diseases version 10 code

^g^As main or side diagnosis in specialised healthcare. ‘No diagnosed mental disorder’ category is not presented

^h^Measured as any inpatient or specialised outpatient healthcare due to any somatic diagnosis (any ICD-10 code except ‘F’, ‘O’, ‘P’ and ‘Q’ codes) during the 3 years before index attempt. ‘No history of specialised healthcare use due to somatic diagnoses’ category is not presented

^i^Measured during the year before index attempt. ‘No neuroleptic drug use’, ‘No anxiolytic drug use’, ‘No hypnotic and sedative drug use’ and ‘No antidepressant drug use’ categories are not presented

^j^Anatomical Therapeutic Chemical classification system code

### Statistical analyses

Differences in the distributions of the covariates among refugees and the Swedish-born were tested using the Chi-square (χ^2^) test. Group-based trajectory modelling [25, 26] was used to estimate trajectory groups of antidepressant use during Y-3 to Y + 3, in separate models among refugees and Swedish-born. Bayesian information criterion (BIC) was used to identify the best-fitted model. In addition to BIC values, a requirement of at least 4% of the study population in each of the subgroups was introduced for model selection to ensure adequate statistical power in the subsequent logistic regression analysis. Probability of an individual belonging to a specific trajectory group was calculated and the highest estimated probability was considered for group belonging. Here, an average probability of ≥0.70 for individuals of a trajectory group, as suggested by Cote and co-authors, was used as an indication for the goodness of fit [25].

We used multinomial logistic regression to test the associations among the trajectory groups and the covariates. Log likelihood χ^2^ tests were applied to estimate if the covariates were associated with a specific trajectory group. Nagelkerke pseudo R^2^ values were applied to evaluate the strength of such potential associations by comparing the full model with the model without the specific covariate. Moreover, to ensure comparability between refugees and the Swedish-born, separate models with or without migration-related factors were analysed. In case of death or emigration during a specific year of follow-up, outcome data for an individual was considered as missing for that year and the subsequent follow-up years. A sensitivity analysis, using forward imputation of annual DDDs of antidepressants from the latest available follow-up year was done to check if our results were biased due to attrition. Data analysis was performed using SAS v. 9.4 (“Proc Traj” was used for the group-based trajectory analysis) [27].

## Results

### Socio-demographic, labour market marginalisation and clinical factors

There were higher proportions of younger people (25–44 years), individuals living in big cities and lower proportion of single people among refugees than the Swedish-born (Table 1). More refugees had long-term unemployment (> 180 days), but a lower proportion of refugees had long-term sickness absence (> 90 net days) or received disability pension during the baseline year, compared with the Swedish-born (Table 1). Among refugees and the Swedish-born, 2.9 and 6.6% respectively, had a history of at least one specialised healthcare contact due to suicide attempt during the 3 years before the index attempt. More refugees had a diagnosis of a depressive disorder or PTSD at index attempt than the Swedish-born (Table 1). A lower proportion of refugees was using anxiolytic, hypnotic or sedative drug(s) during the baseline year, compared with the Swedish-born (Table 1). Differences between refugees and the Swedish-born individuals regarding all socio-demographic, labour market marginalisation and clinical factors were statistically significant based on χ2 tests (*p* < 0.05).

### Trajectory groups in refugees

**Fig. 1:**
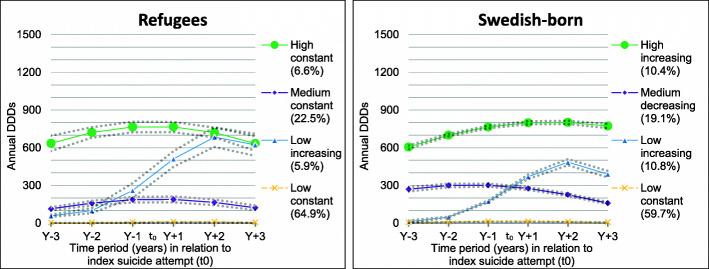
Trajectory groups of antidepressant use according to annual defined daily doses (DDDs) during 3 years before and 3 years after the date of seeking inpatient or specialised outpatient healthcare due to a suicide attempt (t_0_) in between 2009 and 2015 in Sweden among 3492 refugees and 58,950 Swedish-born, aged 20–64 (The dotted lines represent 95% confidence intervals)

The four identified trajectory groups among refugees were labelled as ‘Low constant’, ‘Low increasing’, ‘Medium constant’, and ‘High constant’ (Fig. 1). Most refugees (64.9%) belonged to the ‘Low constant’ group. A sharp increase of annual DDDs was observed among the ‘Low increasing’ group (5.9%) which increased from 60 to 100 DDDs in Y-3 and Y-2 to 620–685 DDDs in Y + 2 and Y + 3. The ‘Medium constant’ (22.5%) and ‘High constant’ (6.6%) groups had 110–190 and 630–765 annual DDDs, respectively during all the observation years.

The distributions and associations of socio-demographic, labour market marginalisation and clinical factors across the four identified trajectory groups among refugees are presented in Table 2. According to the Nagelkerke pseudo R^2^ value, 43.3% of the variance across these trajectory groups was explained by these factors included in the full model. Any use of hypnotic and sedative drugs during the baseline year was estimated to explain 4% of the variance alone (difference in pseudo R^2^ = 0.039), the highest among the individual factors. A 2% estimated difference in pseudo R^2^ was found for each of the following factors: any use of neuroleptic or anxiolytic drugs during the year before index attempt and method of index attempt. The rest of the individual factors had no or minimal influential effect on the full model (difference in pseudo R^2^ = ≤0.01). For all trajectory groups among refugees, the mean probability was ≥0.88, showing good model fit [25].

**Table 2:** Distributions and associations of covariates in each trajectory group of antidepressant use among refugees^a^

Characteristics	Low constant n (column %)	Low increasing n (column %)	Medium constant n (column %)	High constant n (column %)	*p*-value of log-likelihood χ^2^ test	Difference^b^ in Nagelkerke pseudo R^2^
All (row percentage)	2267 (64.9%)	207 (5.9%)	786 (22.5%)	232 (6.6%)		
*Socio-demographic factors*^*c*^						
Sex						
Women	887 (39.1)	110 (53.1)	440 (56.0)	136 (58.6)	< 0.0001	0.008
Men	1380 (60.9)	97 (46.9)	346 (44.0)	96 (41.4)		
Age (years)						
20–24	474 (20.9)	16 (7.7)	87 (11.1)	< 10^d^	< 0.0001	0.014
25–34	726 (32.0)	48 (23.2)	218 (27.7)	36 (15.5)		
35–44	531 (23.4)	68 (32.9)	208 (26.5)	58 (25.0)		
45–54	369 (16.3)	61 (29.5)	196 (24.9)	88 (37.9)		
55–64	167 (7.4)	14 (6.8)	77 (9.8)	47 (20.3)		
Educational level (years)						
Compulsory school (0–9)	810 (35.7)	60 (29.0)	283 (36.0)	68 (29.3)	0.47	0.001
High school (10–12)	870 (38.4)	99 (47.8)	289 (36.8)	100 (43.1)		
College or university (> 12)	518 (22.8)	40 (19.3)	191 (24.3)	59 (25.4)		
Missing	69 (3.0)	< 10^d^	23 (2.9)	<10^d^		
Family situation						
Married/cohabiting without children living at home	221 (9.7)	21 (10.1)	84 (10.7)	40 (17.2)	0.12	0.001
Married/cohabiting with children living at home	616 (27.2)	68 (32.9)	202 (25.7)	62 (26.7)		
Single/divorced/separated/widowed without children living at home	1246 (55.0)	96 (46.4)	411 (52.3)	107 (46.2)		
Single/divorced/separated/widowed with children living at home	184 (8.1)	22 (10.6)	89 (11.3)	23 (9.9)		
Type of residential area^e^						
Big cities	1097 (48.4)	99 (47.8)	378 (48.1)	131 (56.5)	0.28	0.001
Medium-sized cities	767 (33.8)	71 (34.3)	268 (34.1)	67 (28.9)		
Small cities/villages	403 (17.8)	37 (17.9)	140 (17.8)	34 (14.7)		
*Labour market marginalisation factors*^*f*^						
Unemployed, 1–180 days	542 (23.9)	36 (17.4)	157 (20.0)	35 (15.1)	0.34	0.001
Unemployed, > 180 days	208 (9.2)	29 (14.0)	74 (9.4)	15 (6.5)		
Sickness absence, 1–90 net days	135 (6.0)	18 (8.7)	73 (9.3)	16 (6.9)	< 0.0001	0.007
Sickness absence, > 90 net days	70 (3.1)	30 (14.5)	104 (13.2)	36 (15.5)		
Disability pension	159 (7.0)	43 (20.8)	141 (17.9)	94 (40.5)	< 0.001	0.004
*Clinical factors*						
History of any suicide attempt^g^	51 (2.2)	<10^d^	30 (3.8)	13 (5.6)	0.48	0.001
Method of index attempt (ICD-10 code^h^)						
Self-poisoning (X60–69, Y10–19)	894 (39.4)	162 (78.3)	554 (70.5)	183 (78.9)	< 0.0001	0.020
Self-injury (X70–84, Y20–34)	1373 (60.6)	45 (21.7)	232 (29.5)	49 (21.1)		
Mental disorder^i^ at index attempt (ICD-10 code^h^)						
Depressive disorders (F32-F34)	67 (3.0)	35 (16.9)	104 (13.2)	43 (18.5)	0.09	0.001
Bipolar disorders (F30-F31)	<10^d^	<10^d^	<10^d^	<10^d^		
Anxiety disorders (F38-F48 except F43.1)	84 (3.7)	13 (6.3)	50 (6.4)	22 (9.5)		
Post-traumatic stress disorder (F43.1)	<10^d^	<10^d^	21 (2.7)	<10^d^		
Schizophrenia, schizotypal and delusional disorder (F20-F29)	19 (0.8)	<10^d^	16 (2.0)	<10^d^		
Other mental disorders (F01-F19, F50-F99)	121 (5.3)	17 (8.2)	77 (9.8)	22 (9.5)		
History of specialised healthcare use due to somatic diagnoses^j^	1619 (71.4)	174 (84.1)	663 (84.4)	202 (87.1)	0.02	0.002
Use of psychotropic drug(s)^k^ except antidepressants (ATC code^l^)						
Neuroleptic drug(s) (N05A)	92 (4.1)	73 (35.3)	211 (26.8)	90 (38.8)	< 0.0001	0.019
Anxiolytic drug(s) (N05B)	205 (9.0)	100 (48.3)	307 (39.1)	139 (59.9)	< 0.0001	0.019
Hypnotic and sedative drug(s) (N05C)	262 (11.6)	145 (70.0)	417 (53.1)	173 (74.6)	< 0.0001	0.039

^a^Trajectory group of antidepressant use according to annual defined daily doses (DDDs) among 3492 refugees, aged 20–64 years and residing in Sweden who sought inpatient or specialised outpatient healthcare for the index attempt in between 2009 and 2015

^b^Difference in Nagelkerke pseudo R^2^ between model including tested variable and model without tested variable. Nagelkerke pseudo R^2^ for full model including all socio-demographic, labour market marginalisation and clinical factors is 0.433

^c^All socio-demographic factors were measured during the year before index attempt except sex and age which were measured at the index attempt

^d^For ethical reasons i.e. to ensure anonymity, if the number is <10, it is not reported

^e^Type of residential area: big cities - Stockholm, Gothenburg and, Malmö; medium-sized cities - cities with more than 90,000 inhabitants within 30 km distance from the centre of the city; small cities/villages

^f^All labour market marginalisation factors were measured during the year before index attempt. ‘No unemployment’, ‘No sickness absence’ and ‘No disability pension’ categories are not presented

^g^Measured as any inpatient or specialised outpatient healthcare due to suicide attempt during the 3 years before index attempt

^h^International Classification of Diseases version 10 code

^i^As main or side diagnosis in specialised healthcare. ‘No diagnosed mental disorder’ category is not presented

^j^Measured as any inpatient or specialised outpatient healthcare due to a somatic diagnosis (any ICD-10 code except ‘F’, ‘O’, ‘P’ and ‘Q’ codes) during the 3 years before index attempt. ‘No history of specialised healthcare use due to somatic diagnoses’ category is not presented

^k^Measured during the year before index attempt. ‘No neuroleptic drug use’, ‘No anxiolytic drug use’ and ‘No hypnotic and sedative drug use’ categories are not presented

^l^Anatomical Therapeutic Chemical classification system code

There were more women in all the groups except for the ‘Low constant’, where 60.9% were men. The ‘Low constant’ group was mainly composed of younger people and mostly older individuals comprised the ‘High constant’ group (Table 2). The proportion of individuals with sickness absence or disability pension in the ‘Low constant’ group was much lower than in the rest of the groups (Table 2). Almost equal distributions of other sociodemographic and labour market marginalisation factors were observed across the four trajectory groups (Table 2).

Regarding the method of index attempt, the ‘Low constant’ group had 39.4% individuals who used self-poisoning whereas the other groups had 70.5–78.9% individuals using this method. The proportion of individuals who used neuroleptic or anxiolytic drugs during the year before the index attempt in the ‘Low constant’ group was 4.1 and 9%, respectively; the corresponding figures for the rest of the trajectory groups were more than four times higher (Table 2). Almost three-fourths of the ‘High constant’ group used any hypnotic and sedative drug during the year before index attempt, compared with only 11.6% among the ‘Low constant’ group. The distributions of other clinical factors were fairly similar across the four trajectory groups (Table 2).

A regression model for refugees including the migration-related factors (country of birth and duration of residence) explained 43.9% of the variability (see Supplementary Table S1 in Additional file 1) which was comparable with the model without these factors that explained 43.3% of the variability (Table 2). Both factors were fairly evenly distributed among the trajectory groups except there were 2–3 times more refugees from Iran in the ‘High constant’ group than in the other groups (see Supplementary Table S1 in Additional file 1).

### Trajectory groups in the Swedish-born

Among the Swedish-born, four trajectory groups were identified. The DDD levels, proportions and trends, however, were slightly different than those identified among refugees (Fig. 1). Compared to their refugee counterparts, the Swedish-born had a slightly lower proportion of individuals in the ‘Low constant’ group (59.7%). In the ‘Low increasing’ group (10.8%), a steady increase of annual DDDs was observed, from 10 to 50 DDDs in Y-3 and Y-2 to around 500 DDDs in Y + 2. This increase of annual DDDs was much sharper in the same group in refugees (Fig. 1). The patterns of the trajectory groups ‘Medium decreasing’ (19.1%) and ‘High increasing’ (10.4%) among the Swedish-born were somewhat different than the ‘Medium constant’, and ‘High constant’ trajectory groups among refugees (Fig. 1), but proportions were fairly comparable. For all trajectory groups among the Swedish-born, the mean probability was ≥0.92, showing good model fit [25].

The full model including all covariates for the Swedish-born explained 38.9% of the variance across the four identified trajectory groups (Table 3, Nagelkerke pseudo R^2^ value = 0.389). The biggest differences between the Swedish-born and refugees included a lower variance for previous use of hypnotic and sedative drugs and any use of neuroleptic drugs during the year before index attempt having no effect on the model for the Swedish-born (Table 3). The other individual factors in the model for Swedish-born had somewhat similar distributions across the trajectory groups and had similar influence on the full model according to the difference in pseudo R^2^ (Table 3), compared with the model for refugees (Table 2).

**Table 3:** Distributions and associations of covariates in each trajectory group of antidepressant use among Swedish-born^a^

Characteristics	Low constant n (column %)	Low increasing n (column %)	Medium decreasing n (column %)	High increasing n (column %)	*p*-value of log-likelihood χ^2^ test	Difference^b^ in Nagelkerke pseudo R^2^
All (row percentage)	35,205 (59.7)	6340 (10.8)	11,265 (19.1)	6140 (10.4)		
*Socio-demographic factors*^c^						
Sex						
Women	13,449 (38.2)	3595 (56.7)	6809 (60.4)	4105 (66.9)	< 0.0001	0.013
Men	21,756 (61.8)	2745 (43.3)	4456 (39.6)	2035 (33.1)		
Age (years)						
20–24	8774 (24.9)	1801 (28.4)	2122 (18.8)	654 (10.7)	< 0.0001	0.002
25–34	8300 (23.6)	1637 (25.8)	2897 (25.7)	1300 (21.2)		
35–44	6399 (18.2)	1089 (17.2)	2388 (21.2)	1402 (22.8)		
45–54	6513 (18.5)	1116 (17.6)	2285 (20.3)	1634 (26.6)		
55–64	5219 (14.8)	697 (11.0)	1573 (14.0)	1150 (18.7)		
Educational level (years)						
Compulsory school (0–9)	8503 (24.2)	1725 (27.2)	3370 (29.9)	1628 (26.5)	< 0.0001	0.001
High school (10–12)	19,487 (55.4)	3430 (54.1)	5722 (50.8)	3191 (52.0)		
College or university (> 12)	6904 (19.6)	1104 (17.4)	2041 (18.1)	1246 (20.3)		
Missing	311 (0.9)	81 (1.3)	132 (1.2)	75 (1.2)		
Family situation						
Married/cohabiting without children living at home	3893 (11.1)	525 (8.3)	1020 (9.1)	762 (12.4)	< 0.01	< 0.001
Married/cohabiting with children living at home	6470 (18.4)	897 (14.1)	1382 (12.3)	795 (12.9)		
Single/divorced/separated/widowed without children living at home	23,212 (66.0)	4401 (69.4)	7862 (69.7)	4058 (66.1)		
Single/divorced/separated/widowed with children living at home	1630 (4.6)	517 (8.2)	1001 (8.9)	525 (8.6)		
Type of residential area^d^						
Big cities	11,158 (31.7)	2020 (31.9)	3529 (31.3)	1973 (32.1)	0.36	< 0.001
Medium-sized cities	12,215 (34.7)	2427 (38.3)	4346 (38.6)	2407 (39.2)		
Small cities/villages	11,832 (33.6)	1893 (29.9)	3390 (30.1)	1760 (28.7)		
*Labour market marginalisation factors*^c, e^						
Unemployed, 1–180 days	5648 (16.0)	1148 (18.1)	1949 (17.3)	741 (12.1)	< 0.0001	< 0.001
Unemployed, > 180 days	1225 (3.5)	236 (3.7)	420 (3.7)	134 (2.2)		
Sickness absence, 1–90 net days	2989 (8.5)	856 (13.5)	1411 (12.5)	699 (11.4)	< 0.0001	0.013
Sickness absence, > 90 net days	1441 (4.1)	598 (9.4)	1966 (17.5)	1262 (20.6)		
Disability pension	4261 (12.1)	1003 (15.8)	3352 (29.8)	2716 (44.2)	< 0.0001	0.009
*Clinical factors*						
History of any suicide attempt^f^	1404 (4.0)	364 (5.7)	1329 (11.8)	771 (12.6)	< 0.0001	0.002
Method of index attempt (ICD-10 code^g^)						
Self-poisoning (X60–69, Y10–19)	11,782 (33.5)	4339 (68.4)	7779 (69.1)	4465 (72.7)	< 0.0001	0.024
Self-injury (X70–84, Y20–34)	23,423 (66.5)	2001 (31.6)	3486 (30.9)	1675 (27.3)		
Mental disorder^h^ at index attempt (ICD-10 code^g^)						
Depressive disorders (F32-F34)	813 (2.3)	684 (10.8)	967 (8.6)	700 (11.4)	< 0.0001	0.001
Bipolar disorders (F30-F31)	226 (0.6)	89 (1.4)	316 (2.8)	184 (3.0)		
Anxiety disorders (F38-F48 except F43.1)	919 (2.6)	521 (8.2)	795 (7.1)	536 (8.7)		
Post-traumatic stress disorder (F43.1)	21 (0.1)	19 (0.3)	25 (0.2)	16 (0.3)		
Schizophrenia, schizotypal and delusional disorder (F20-F29)	252 (0.7)	76 (1.2)	131 (1.2)	98 (1.6)		
Other mental disorders (F01-F19, F50-F99)	3683 (10.5)	1144 (18.0)	2288 (20.3)	1145 (18.6)		
History of specialised healthcare use due to somatic diagnoses^i^	25,401 (72.2)	4912 (77.5)	9446 (83.9)	5189 (84.5)	< 0.001	< 0.001
Use of psychotropic drug(s)^j^ except antidepressants (ATC code^k^)						
Neuroleptic drug(s) (N05A)	1982 (5.6)	1040 (16.4)	3285 (29.2)	2327 (37.9)	< 0.0001	0.010
Anxiolytic drug(s) (N05B)	4899 (13.9)	3023 (47.7)	6088 (54.0)	4095 (66.7)	< 0.0001	0.023
Hypnotic and sedative drug(s) (N05C)	5897 (16.8)	3112 (49.1)	6832 (60.6)	4342 (70.7)	< 0.0001	0.019

^a^Trajectory group of antidepressant use according to annual defined daily doses (DDDs) among 58,950 Swedish-born, aged 20–64 years and residing in Sweden who sought inpatient or specialised outpatient healthcare for the index attempt in between 2009 and 2015

^b^Difference in Nagelkerke pseudo R^2^ between model including tested variable and model without tested variable. Nagelkerke pseudo R2 for full model including all socio-demographic, labour market marginalisation and clinical factors is 0.389

^c^All socio-demographic factors were measured during the year before index attempt except sex and age which were measured at the index attempt

^d^Type of residential area: big cities - Stockholm, Gothenburg and, Malmö; medium-sized cities - cities with more than 90,000 inhabitants within 30 km distance from the centre of the city; small cities/villages

^e^All labour market marginalisation factors were measured during the year before index attempt. ‘No unemployment’, ‘No sickness absence’ and ‘No disability pension’ categories are not presented

^f^Measured as any inpatient or specialised outpatient healthcare due to suicide attempt during the 3 years before index attempt

^g^International Classification of Diseases version 10 code

^h^As main or side diagnosis in specialised healthcare. ‘No diagnosed mental disorder’ category is not presented

^i^Measured as any inpatient or specialised outpatient healthcare due to a somatic diagnosis (any ICD-10 code except ‘F’, ‘O’, ‘P’ and ‘Q’ codes) during the 3 years before index attempt. ‘No history of specialised healthcare use due to somatic diagnoses’ category is not presented

^j^Measured during the year before index attempt. ‘No neuroleptic drug use’, ‘No anxiolytic drug use’ and ‘No hypnotic and sedative drug use’ categories are not presented

^k^Anatomical Therapeutic Chemical classification system code

### Sensitivity analyses

In both sensitivity analysis 1 (where suicide attempt cases with undetermined intent were excluded) and 2 (where suicide attempters who were treated in specialised outpatient healthcare were excluded), the patterns (data not shown) of the trajectory groups among refugees and the Swedish-born were the same as in the main analyses. Regarding group belonging, the ‘Low constant’ group in both sensitivity analyses consisted of around 10% lower proportions of refugees and Swedish-born, compared with the main analyses (data not shown). Differences among the other trajectory groups identified in the sensitivity analyses and the trajectory groups identified in our main analysis were marginal (data not shown). In sensitivity analysis 3 where missing outcome data due to death or emigration was imputed, similar trajectory groups were identified among refugees and the Swedish-born (data not shown), compared with our main analysis. A fourth sensitivity analysis, restricting the cohort to only suicide attempters who had a diagnosis of depressive disorders at index attempt (*n* = 249 refugees, 3164 Swedish-born) yielded similar results to our main analysis (Data not shown). Finally, when antidepressant use was measured 6-monthly, the patterns (supplementary figure 1 in Additional file 1) of the trajectory groups among refugees and the Swedish-born were almost the same as in the main analyses with yearly follow-up data. Furthermore, including all individuals in the cohort in a single model, instead of separate models for refugees and the Swedish-born, we could see that the patterns and composition of the trajectory groups identified in this single model were almost the same as the patterns and composition of the trajectory groups among the Swedish-born (supplementary figure 1 in Additional file 1). When testing the associations among the trajectory groups found in this single model and the covariates, we also entered refugee status (‘Swedish-born’ = 0 and ‘Refugee’ = 1) as a separate covariate in the multinomial logistic regression analysis. The difference in nagelkerke pseudo R^2^ values between a full model and a model without refugee status showed that this variable was not influential (difference in pseudo R^2^ 0.001, data not shown).

## Discussion

### Main findings

In this population-based longitudinal study, four different trajectories of antidepressant use during 3 years before and after a suicide attempt were identified among all 3492 refugees and 58,950 Swedish-born individuals, respectively, who were 20–64-years-old when receiving specialised healthcare due to suicide attempt during 2009–2015 in Sweden. During this period, antidepressant use was constantly low (≤15 DDDs) for 65% of refugees. Among 6% of refugees, a low antidepressant use before a suicide attempt (< 100 DDDs) sharply increased after the attempt (around 650 DDDs). Two other trajectory groups had constant use of antidepressants at medium and high levels (22.5 and 6.6% of refugees had 110–190 and 630–765 DDDs, respectively). The method of index attempt and any use of psychotropic drugs during the year before index attempt influentially determined the differences among the trajectory groups in refugees. The patterns and composition of the trajectory groups and the association of these trajectories with the covariates among the Swedish-born were fairly similar to those among refugees.

The majority among refugees and the Swedish-born suicide attempters belonged to the ‘Low constant’ trajectory of antidepressant use. Although ‘low use’ was more prevalent among refugees (64.9%) than the Swedish-born (59.7%), the difference was quite small between these groups. The discrepancies in proportions of other psychotropic drug use among refugees and the Swedish-born during the year before suicide attempt were also marginal. It is not possible to make a direct comparison of these results with the literature because, to our knowledge, no previous studies investigated such trajectories before and after a suicide attempt. In a cross-sectional study in 2009, the prevalence of antidepressant use among refugees was considerably lower in refugees than in Swedish-born [28]. Much stronger differences between refugees and Swedish-born were found in a study focusing on young individuals with common mental disorders which reported considerably lower initiation of antidepressants in young refugees than their Swedish-born peers [29]. These contrasting findings regarding the degree of difference in antidepressant treatment in refugees, compared to host population, might be due to differences in study populations i.e. if the general population is investigated or different diagnostic groups. Also, discrepancies in socio-demographic factors (e.g. age group and socioeconomic status), as well as health status at baseline, might underlie the observed differences.

Some marginal differences were seen between refugees and the Swedish-born concerning the ‘Low increasing’ trajectories. There was a slightly lower proportion of refugees (5.9%) than the Swedish-born (10.8%) who belonged to these trajectory groups. Also, the increase of annual DDDs of antidepressants after the index attempt was much sharper among refugees than the Swedish-born. A possible explanation for this sharper increase in refugees than the Swedish-born can be that refugees in this group had a higher medical severity at baseline or received inadequate treatment preceding the attempt, which was then followed by higher dosages and adequate treatment with antidepressants after the attempt. Future studies should investigate if other factors, such as a higher number of reattempts among refugees who belonged to this trajectory group, can explain these findings.

Comparing the ‘Low constant’ and ‘Low increasing’ trajectories between refugees and the Swedish-born reveals that, in general, proportions of refugee suicide attempters using antidepressants were somewhat lower than that among the Swedish-born. This is in line with previous research that reported lower psychiatric healthcare use [9, 30] and treatment [29] in refugees than the host population. On the one hand, this may suggest that refugees have unmet needs for the treatment of their mental ill-health. On the other hand, lower use of antidepressants can be due to the side-effects of medication or mistrust in Western medicine. Furthermore, socio-cultural biases such as lack of proficiency in the Swedish language could have hampered expressing their mental distresses and therefore, they received fewer prescriptions. There can also be cultural differences in the expression of symptoms of mental ill-health leading to under-diagnosis and management. Higher levels of stigma towards mental ill-health and somatization of symptoms of underlying mental disorder may further contribute to such under-diagnosis and treatment. Furthermore, due to cultural influences, refugees may prefer alternative medicine like herbal remedies etc. over pharmacotherapy for treatment of mental disorders [31].

Considering so many potential barriers to healthcare access for refugees, the fact that we found only marginal differences in antidepressant use related to the ‘Low constant’ and ‘Low increasing’ trajectories and hardly any differences related to the two other trajectory groups at ‘Medium’ and ‘High’ levels between refugee and Swedish-born suicide attempters was somewhat surprising. Reasons for these comparable patterns might be due to the Swedish healthcare system managing quite well in bridging the treatment gap between refugees and Swedish-born particularly when it comes to suicide attempters. An alternative explanation might, however, be that refugees have a higher medical severity when they get specialised healthcare due to a suicide attempt due to the known barriers to specialised healthcare. This in turn might explain the comparable treatment rates to Swedish-born, i.e. relatively higher rates than what would be expected. Our data might not be sufficient to test this hypothesis as we don’t have access to information on the medical severity of the underlying disease or the severity of the suicide attempt. Further studies with information on suicide attempters with such clinical data are warranted to elucidate these associations. Finally, a third potential explanation for the apparent similarities rather than differences between the trajectory groups of antidepressant use among refugees and the Swedish-born could be due to the fact that most refugees (87%) in our cohort had been living in Sweden for longer than 5 years and a longer duration of residence was reported to be favourable for increasing access and use of psychiatric healthcare [28].

### Association of covariates with identified trajectory groups

The socio-demographic, labour market marginalisation and clinical factors included in the full model explained around 43 and 39% of the variance across the trajectory groups among refugees and Swedish-born, respectively. It may suggest that, in determining the trajectory group belonging for refugees and the Swedish-born, there could be cultural and other unmeasured factors which will be worthwhile to investigate in future studies.

Any use of anxiolytic or hypnotic and sedative drugs during the year before index attempt was the most influential clinical factors in explaining the variability among the trajectory groups among refugees and the Swedish-born. The use of other psychotropic medication than antidepressants before the index attempt might here reflect a better knowledge and acceptance of the healthcare system or higher medical severity of the underlying mental disorder. We found that the difference in Nagelkerke pseudo R^2^ related to the use of hypnotics and sedatives was higher for refugees than in Swedish-born. This difference in pseudo R^2^ is based on a more skewed distribution across the trajectory groups in refugees than the Swedish-born; the biggest difference being among the ‘Low increasing’ trajectory group. This trajectory group also showed the biggest differences in temporal DDD level changes between refugees and Swedish-born and might, as previously mentioned, reflect differences regarding the medical severity.

### Strengths and limitations

To the best of our knowledge, this is the first study where trajectories of antidepressant use before and after a suicide attempt among refugees and Swedish-born are explored. The main strength of this study is the population-based cohort design which allowed adequate statistical power for group-based trajectory analyses among a minority group i.e. refugee suicide attempters. Another strength is the use of high-quality [16–20] nationwide register data on DDDs of antidepressants and several covariates which also limited the possibility of recall bias and selection bias from non-response.

Our results should be interpreted within the context of some limitations. First, while previous surveys showed that approximately 50% of suicide attempters in Sweden require hospitalisation [32], the available data allowed only the inclusion of suicide attempters treated in specialised healthcare in this study. For refugees, this may have led to differential selection into the study population, because they probably had underreported suicidal behaviour differently than the Swedish-born population, due to higher levels of stigma associated with this behaviour [7]. Although this differential selection may have hampered the generalisability of our results, we believe that we were able to minimize this bias in both groups by including the events of undetermined intent (ICD-10 codes: Y10–Y34) as suicide attempts. Second, for 0.02% of refugees and Swedish-born individuals, the annual measure of use of antidepressants lacked data for the last 2 months during Y + 3. We think, though, that this has not affected our results. Moreover, the DDDs of antidepressants registered as purchases may not indicate the actual use of antidepressants. In Sweden, register data on antidepressants include prescriptions from primary and specialised outpatient healthcare, but not hospitalised care, suggesting some underestimation in our study. Furthermore, underuse in the form of non-compliance after purchase may occur and some individuals may overuse by obtaining unregistered drugs from abroad or via the internet. Also, we did not have information on the clinical indication for using an antidepressant. Antidepressants can be prescribed for other reasons than common mental disorders e.g. chronic pain. However, these conditions are often co-morbid and idioms of distress may present as somatic symptoms in refugees [33]. Finally, generalisability of our results to refugees, who arrived recently in a host country, may have been compromised because most individuals (87%) in this cohort of refugee suicide attempters had already been living in Sweden for longer than 5 years when they entered the cohort. Moreover, these results are not generalisable to asylum seekers awaiting legal status as refugees or to individuals living in refugee camps or in countries with substantially different healthcare systems than Sweden.

## Conclusion

The diagnostic profile related to depressive and anxiety disorders typically treated with antidepressants did not considerably differ in refugee and Swedish-born suicide attempters. In general, patterns and characteristics of antidepressant drug use in these two groups were comparable, but somewhat fewer refugees than Swedish-born were prescribed antidepressants. This was also the case for anxiolytics, hypnotics and sedatives. Despite previous reports on refugees in the general population being considerably undertreated regarding psychiatric care, this study revealed some but not major differences in antidepressant treatment between refugees and Swedish-born individuals, 3 years before and after a suicide attempt.

## Supplementary Information

**Additional file 1. **A. Distributions and associations of covariates including migration-related characteristics among refugees and B. Trajectory groups of antidepressant use according to 6-monthly defined daily doses (DDDs). A. Distributions and associations of socio-demographic, labour market marginalisation, clinical *and migration-related characteristics* in each trajectory group of antidepressant use according to annual defined daily doses (DDDs) among 3492 refugees, aged 20–64 years and residing in Sweden who sought inpatient or specialised outpatient healthcare for suicide attempt (index attempt) in between 2009 and 2015. Includes results from the supplementary analysis for refugees including migration-related factors which were not applicable for the Swedish-born and therefore, those were not included in our main analysis. B. Trajectory groups of antidepressant use according to 6-monthly defined daily doses (DDDs) during 3 years before and 3 years after the date of seeking inpatient or specialised outpatient healthcare due to a suicide attempt (t0) in between 2009 and 2015 in Sweden among 3492 refugees, 58,950 Swedish-born and the whole cohort, aged 20–64 (The dotted lines represent 95% confidence intervals).

## Data Availability

The register data used in this study contain sensitive information at an individual level and therefore, are not publicly available due to confidentiality. Data is available on request for any interested researchers to allow replication of results through the National Data Services in Sweden, provided all ethical and legal requirements are met. Detailed information on data application in Sweden can be found at ‘https://www.registerforskning.se/en/’.

## Abbreviations

ATC: Anatomical Therapeutic Chemical classification system
BIC: Bayesian Information Criterion
CI: Confidence Interval
DDD: Defined Daily Dose
ICD-10: International Classification of Diseases version 10
PTSD: Post-Traumatic Stress Disorder
