# Supplementary material for: Trajectories of antidepressant use before and after a suicide attempt among refugees and Swedish-born individuals: a cohort study
Source: Int J Equity Health. 2021 Jun 2;20:131. doi: 10.1186/s12939-021-01460-z (PMC8170815; doi:10.1186/s12939-021-01460-z)
Supplement: Supplementary file 1 — Additional file 1. A. Distributions and associations of covariates including migration-related characteristics among refugees and B. Trajectory groups of antidepressant use according to 6-monthly defined daily doses (DDDs). A. Distributions and associations of socio-demographic, labour market marginalisation, clinical and migration-related characteristics in each trajectory group of antidepressant use according to annual defined daily doses (DDDs) among 3492 refugees, aged 20–64 years and residing in Sweden who sought inpatient or specialised outpatient healthcare for suicide attempt (index attempt) in between 2009 and 2015. Includes results from the supplementary analysis for refugees including migration-related factors which were not applicable for the Swedish-born and therefore, those were not included in our main analysis. B. Trajectory groups of antidepressant use according to 6-monthly defined daily doses (DDDs) during 3 years before and 3 years after the date of seeking inpatient or specialised outpatient healthcare due to a suicide attempt (t0) in between 2009 and 2015 in Sweden among 3492 refugees, 58,950 Swedish-born and the whole cohort, aged 20–64 (The dotted lines represent 95% confidence intervals). [file 12939_2021_1460_MOESM1_ESM.docx]

**Supplementary Table S1:** Distributions and associations of covariates including migration-related characteristics among refugees^a^

| Characteristics | Low constant  n (column %) | Low increasing  n (column %) | Medium constant n (column %) | High  constant  n (column %) | p-value of log-likelihood χ^2^ test | Difference^b^ in Nagelkerke pseudo R^2^ |
| --- | --- | --- | --- | --- | --- | --- |
| All (row percentage) | 2 267 (64.9%) | 207 (5.9%) | 786 (22.5%) | 232 (6.6%) |  |  |
| *Socio-demographic factors^c^* |  |  |  |  |  |  |
| Sex |  |  |  |  |  |  |
| Women | 887 (39.1) | 110 (53.1) | 440 (56.0) | 136 (58.6) | <0.0001 | 0.008 |
| Men | 1 380 (60.9) | 97 (46.9) | 346 (44.0) | 96 (41.4) |  |  |
| Age (years) |  |  |  |  |  |  |
| 20–24 | 474 (20.9) | 16 (7.7) | 87 (11.1) | <10^d^ | <0.0001 | 0.013 |
| 25–34 | 726 (32.0) | 48 (23.2) | 218 (27.7) | 36 (15.5) |  |  |
| 35–44 | 531 (23.4) | 68 (32.9) | 208 (26.5) | 58 (25.0) |  |  |
| 45–54 | 369 (16.3) | 61 (29.5) | 196 (24.9) | 88 (37.9) |  |  |
| 55–64 | 167 (7.4) | 14 (6.8) | 77 (9.8) | 47 (20.3) |  |  |
| Educational level (years) |  |  |  |  |  |  |
| Compulsory school (0-9) | 810 (35.7) | 60 (29.0) | 283 (36.0) | 68 (29.3) | 0.35 | 0.001 |
| High school (10-12) | 870 (38.4) | 99 (47.8) | 289 (36.8) | 100 (43.1) |  |  |
| College or university (>12) | 518 (22.8) | 40 (19.3) | 191 (24.3) | 59 (25.4) |  |  |
| Missing | 69 (3.0) | <10^d^ | 23 (2.9) | <10^d^ |  |  |
| Family situation |  |  |  |  |  |  |
| Married/cohabiting without children living at home | 221 (9.7) | 21 (10.1) | 84 (10.7) | 40 (17.2) | 0.11 | 0.001 |
| Married/cohabiting with children living at home | 616 (27.2) | 68 (32.9) | 202 (25.7) | 62 (26.7) |  |  |
| Single/divorced/separated/widowed without children living at home | 1 246 (55.0) | 96 (46.4) | 411 (52.3) | 107 (46.2) |  |  |
| Single/divorced/separated/widowed with children living at home | 184 (8.1) | 22 (10.6) | 89 (11.3) | 23 (9.9) |  |  |
| Type of residential area^e^ |  |  |  |  |  |  |
| Big cities | 1 097 (48.4) | 99 (47.8) | 378 (48.1) | 131 (56.5) | 0.49 | 0.001 |
| Medium-sized cities | 767 (33.8) | 71 (34.3) | 268 (34.1) | 67 (28.9) |  |  |
| Small cities/villages | 403 (17.8) | 37 (17.9) | 140 (17.8) | 34 (14.7) |  |  |
| *Labour market marginalisation factors^f^* |  |  |  |  |  |  |
| Unemployed, 1-180 days | 542 (23.9) | 36 (17.4) | 157 (20.0) | 35 (15.1) | 0.22 | 0.001 |
| Unemployed, >180 days | 208 (9.2) | 29 (14.0) | 74 (9.4) | 15 (6.5) |  |  |
| Sickness absence, 1-90 net days | 135 (6.0) | 18 (8.7) | 73 (9.3) | 16 (6.9) | <0.0001 | 0.007 |
| Sickness absence, >90 net days | 70 (3.1) | 30 (14.5) | 104 (13.2) | 36 (15.5) |  |  |
| Disability pension | 159 (7.0) | 43 (20.8) | 141 (17.9) | 94 (40.5) | <0.01 | 0.003 |
| *Clinical factors* |  |  |  |  |  |  |
| History of any suicide attempt^g^ | 51 (2.2) | <10^d^ | 30 (3.8) | 13 (5.6) | 0.42 | 0.001 |
| Method of index attempt (ICD-10 code^h^) |  |  |  |  |  |  |
| Self-poisoning (X60-69, Y10-19) | 894 (39.4) | 162 (78.3) | 554 (70.5) | 183 (78.9) | <0.0001 | 0.021 |
| Self-injury (X70-84, Y20-34) | 1 373 (60.6) | 45 (21.7) | 232 (29.5) | 49 (21.1) |  |  |
| Mental disorder^i^ at index attempt (ICD-10 code^h^) |  |  |  |  |  |  |
| Depressive disorders (F32-F34) | 67 (3.0) | 35 (16.9) | 104 (13.2) | 43 (18.5) | 0.07 | 0.002 |
| Bipolar disorders (F30-F31) | <10^d^ | <10^d^ | <10^d^ | <10^d^ |  |  |
| Anxiety disorders (F38-F48 except F43.1) | 84 (3.7) | 13 (6.3) | 50 (6.4) | 22 (9.5) |  |  |
| Post-traumatic stress disorder (F43.1) | <10^d^ | <10^d^ | 21 (2.7) | <10^d^ |  |  |
| Schizophrenia, schizotypal and delusional disorder (F20-F29) | 19 (0.8) | <10^d^ | 16 (2.0) | <10^d^ |  |  |
| Other mental disorders (F01-F19, F50-F99) | 121 (5.3) | 17 (8.2) | 77 (9.8) | 22 (9.5) |  |  |
| History of specialised healthcare use due to somatic diagnoses^j^ | 1 619 (71.4) | 174 (84.1) | 663 (84.4) | 202 (87.1) | 0.02 | 0.002 |
| Use of psychotropic drug(s)^k^ except antidepressants (ATC code^l^) |  |  |  |  |  |  |
| Neuroleptic drug(s) (N05A) | 92 (4.1) | 73 (35.3) | 211 (26.8) | 90 (38.8) | <0.0001 | 0.019 |
| Anxiolytic drug(s) (N05B) | 205 (9.0) | 100 (48.3) | 307 (39.1) | 139 (59.9) | <0.0001 | 0.018 |
| Hypnotic and sedative drug(s) (N05C) | 262 (11.6) | 145 (70.0) | 417 (53.1) | 173 (74.6) | <0.0001 | 0.039 |
| *Migration-related factors^m^* |  |  |  |  |  |  |
| Country of birth |  |  |  |  |  |  |
| Eritrea | 44 (1.9) | <10^d^ | <10^d^ | <10^d^ | 0.11 | 0.001 |
| Ethiopia | 35 (1.5) | <10^d^ | 11 (1.4) | <10^d^ |  |  |
| Somalia | 109 (4.8) | <10^d^ | 21 (2.7) | <10^d^ |  |  |
| Afghanistan | 75 (3.3) | <10^d^ | 26 (3.3) | <10^d^ |  |  |
| Iran | 246 (10.9) | 27 (13.0) | 121 (15.4) | 66 (28.4) |  |  |
| Iraq | 412 (18.2) | 32 (15.5) | 125 (15.9) | 34 (14.7) |  |  |
| Syria | 64 (2.8) | <10 | 22 (2.8) | <10 |  |  |
| Chile | 78 (3.4) | 12 (5.8) | 27 (3.4) | 14 (6.0) |  |  |
| Former Yugoslavia | 471 (20.8) | 51 (24.6) | 184 (23.4) | 45 (19.4) |  |  |
| Duration of residence (years) |  |  |  |  |  |  |
| 0-5 | 312 (13.8) | 16 (7.7) | 125 (15.9) | 11 (4.7) | <0.001 | 0.004 |
| 6-10 | 511 (22.5) | 39 (18.8) | 133 (16.9) | 25 (10.8) |  |  |
| >10 | 1 444 (63.7) | 152 (73.4) | 528 (67.2) | 196 (84.5) |  |  |
| ^a^Distributions and associations of socio-demographic, labour market marginalisation, clinical and migration-related characteristics in each trajectory group of antidepressant use according to annual defined daily doses (DDDs) among 3 492 refugees, aged 20-64 years and residing in Sweden who sought inpatient or specialised outpatient healthcare for suicide attempt (index attempt) in between 2009 and 2015  ^b^Difference in Nagelkerke pseudo R2 between model including tested variable and model without tested variable. Nagelkerke pseudo R2 for full model including all socio-demographic, labour market marginalisation and clinical factors is 0.433.  ^c^All socio-demographic factors were measured during the year before index attempt except sex and age which were measured at the index attempt.  ^d^For ethical reasons i.e. to ensure anonymity, if the number is <10, it is not reported.  ^e^Type of residential area: big cities - Stockholm, Gothenburg and, Malmö; medium-sized cities - cities with more than 90,000 inhabitants within 30 km distance from the centre of the city; small cities/villages.  ^f^All labour market marginalisation factors were measured during the year before index attempt. ‘No unemployment’, ‘No sickness absence’ and ‘No disability pension’ categories are not presented.  ^g^Measured as any inpatient or specialised outpatient healthcare due to suicide attempt during the three years before index attempt.  hInternational Classification of Diseases version 10 code.  ^i^As main or side diagnosis in specialised healthcare. ‘No diagnosed mental disorder’ category is not presented.  ^j^Measured as any inpatient or specialised outpatient healthcare due to a somatic diagnosis (any ICD-10 code except ‘F’, ‘O’, ‘P’ and ‘Q’ codes) during the three years before index attempt. ‘No history of specialised healthcare use due to somatic diagnoses’ category is not presented.  ^k^Measured during the year before index attempt. ‘No neuroleptic drug use’, ‘No anxiolytic drug use’ and ’No hypnotic and sedative drug use’ categories are not presented.  ^l^Anatomical Therapeutic Chemical classification system code  ^m^All migration-related factors were measured during the year before index attempt. | | | | | | |
|  |  |  |  |  |  |  |

| a  t_0_ |
| --- |
| b  t_0_ |
| c  t_0_ |

**Supplementary Fig. 1** Trajectory groups of antidepressant use according to 6-monthly defined daily doses (DDDs) during 3 years before and 3 years after the date of seeking inpatient or specialised outpatient healthcare due to a suicide attempt (t_0_) in between 2009 and 2015 in Sweden among 3,492 refugees^a^, 58,950 Swedish-born^b^ and the whole cohort^c^, aged 20-64 (The dotted lines represent 95% confidence intervals)
